# Superinfection of Rectovaginal Endometriosis: Case Report and Review of the Literature

**DOI:** 10.3390/diagnostics13091514

**Published:** 2023-04-23

**Authors:** Marta Barba, Andrea Morciano, Tomaso Melocchi, Alice Cola, Alessandra Inzoli, Paolo Passoni, Matteo Frigerio

**Affiliations:** 1 Department of Gynecology and Obstetrics, Pelvic Floor Center, Fondazione IRCCS San Gerardo dei Tintori, University of Milano-Bicocca, 20900 Monza, Italy; 2 Department of Gynecology and Obstetrics, Panico Pelvic Floor Center, Pia Fondazione “Cardinale G. Panico”, 73039 Tricase, Italy; 3 Department of Gynecology and Obstetrics, IRCCS San Gerardo dei Tintori, University of Milano-Bicocca, 20900 Monza, Italy; 4 Department of Gynecology and Obstetrics, Fondazione IRCCS San Gerardo dei Tintori, ASST Monza, Ospedale San Gerardo, 20900 Monza, Italy

**Keywords:** endometriosis, chronic pelvic pain, surgery, pelvic floor ultrasound, rectovaginal space, laparoscopy

## Abstract

Background: A peculiar complication of endometriosis is a superinfection. However, the superinfection of extra-ovarian endometriosis is anecdotal, and only a few cases have been described. We wanted to present the first cases of the superinfection of rectovaginal endometriosis and to perform a literature review of the superinfection of extra-ovarian endometriosis. Methods: We present a case of a 24-year-old woman who was referred to our Pelvic Floor Unit for rectal–perineal pain, dyspareunia, and recurrent episodes of dense purulent vaginal discharge for one year, in which the superinfection of rectovaginal endometriosis was diagnosed. Moreover, we performed a systematic search of the literature indexed on PubMed up to 31 January 2023. Results: Laparoscopic drainage was successful in managing this condition. In the literature, clinical presentation and instrumental and microbiological findings are very heterogeneous. However, the gold standard of management is represented by surgical or percutaneous drainage. Conclusions: In the case of a pelvic abscess, the superinfection of endometriosis lesions should be suspected, and this can represent the onset symptom of endometriosis. Ultrasonography may show nodular or flat hypoechoic lesions with hyperechoic debris and peripheral positive color/power Doppler intensities. The goal of management is to drain the abscess, either percutaneously or via traditional surgery, followed by proper hormonal therapy to reduce recurrence.

## 1. Introduction

Endometriosis is a chronic gynecologic disease characterized by endometrial-like tissue outside the uterus [1]. The ectopic endometrial tissue usually is located in the pelvis, and the most common sites are the ovaries, the posterior cul-de-sac, the uterosacral and broad ligaments, and the fallopian tubes [2]. However, it can appear anywhere in the body, such as in the bowel (above all, the sigmoid colon and the appendix), in the diaphragm, and in the pleural cavity [3]. Endometriosis outside the pelvis is called “extragenital” or “extrapelvic” endometriosis [4].

Nowadays, it is still not possible to determine the exact prevalence since there is no noninvasive, reliable method for diagnosing endometriosis, and many patients are asymptomatic. The prevalence of asymptomatic endometriosis is 1–7% in women seeking elective sterilization, up to 60% of women with pelvic pain, and 50–60% of women with pelvic pain and/or infertility [5]. It is estimated that the overall prevalence of endometriosis is nearly 10% [6]. In a review conducted at Glasgow Royal Hospital, the mean age at diagnosis of endometriosis ranged between 25 and 35 years old [7]; it is rare in premenarchal girls, and most cases in women under the age of 20 are associated with Mullerian anomalies (vaginal or cervical obstruction) [8,9]. Endometriosis is associated with pelvic pain and infertility. However, it shows a broad spectrum of clinical signs and manifestations and is likely to progress and recur [10]; it also has adverse effects on the quality of life and poses a significant economic burden [11]. A specific entity is represented by uterine adenomyosis, defined as the presence of endometrial glands and stroma within the myometrium which can cause pelvic pain, infertility, and abnormal uterine bleeding [12]. Adenomyosis and endometriosis often coexist. However, the prevalence of this association is variable in the literature with conflicting results. Endometriosis lesions in the pelvis can be divided into three main types: superficial peritoneal, deep infiltrating, and ovarian [13]. Deep endometriosis is differentiated from superficial peritoneal and ovarian by the presence of endometriosis which invades the muscle of the bowel and occasionally the nerves and lymph nodes as well [14]. Endometriosis outside the pelvis has always been considered rare. However, nowadays, there are a considerable number of cases [15]. Actually, 34 cases of extrapelvic endometriosis were described out of 379 cases of endometriosis in total, which was an 8.9% incidence of extrapelvic endometriosis [7]. Despite an increasing number of reports, there are no guidelines on the diagnosis or management of extra-pelvic endometriosis. The excision of the lesion of extra-pelvic endometriosis is always the treatment of choice. However, it depends on the location of the lesions. When surgery is not feasible, medical therapy is necessary [16].

As endometriosis has a high recurrence rate, surgery is never curative and must be followed by long-term medical therapy. Recurrence is defined as the recurrence of pain (dysmenorrhea, dyschezia, dyspareunia or pelvic pain), as clinical recurrence (with physical examination), as radiological recurrence (MRI or US), as repeated rises in CA125 markers, or as surgically confirmed lesions [17]. Since there are many definitions, it is not easy to define the exact rate of recurrence, which varies in the literature between 0 and 89.6% of cases [17]. When surgery is performed for ovarian endometrioma, surgeons should prefer ovarian cystectomy instead of drainage for the secondary prevention of endometriosis [18]. The recurrence rate can be reduced by prescribing hormonal therapy (oral contraceptives or Levonorgestrel intrauterine device) [19], which is well demonstrated for patients with ovarian endometriomas, while the evidence is weak for patients with deep endometriosis [20].

Endometriosis may lead to different complications, especially during pregnancy, such as the bleeding or rupture of endometriosis lesions, which can lead to bowel perforation; hemoperitoneum; or acute appendicitis; most of them are linked to decidualization [21]. A peculiar complication of endometriosis is the superinfection of endometriosis, which is considered an uncommon event, estimated to occur in 2% of cases of ovarian endometriosis [22]. However, the superinfection of extra-ovarian endometriosis is anecdotal, and only a few cases have been described. Pain and fever represent the most common symptoms, while more specific disorders depend on endometriosis localization [23]. Superinfection has been thought to be related to locally reduced resistance to infection and endometriotic blood products acting as a culture medium [22]. Potential pathogenetic mechanisms involve direct inoculation, ascension via the lower genital tract, translocation from the adjacent bowel, and hematogenous/lymphatic spread [24]. In the case of infected endometriosis, drainage is recommended, either through conventional surgery or interventional radiology [24]. However, neither guidelines nor reviews on this topic exist, thus making it difficult to make recommendations on the management of this rare condition. Consequently, with the present paper, we wanted to present the first cases of the superinfection of rectovaginal endometriosis and to perform a literature review of the superinfection of extra-ovarian endometriosis.

## 2. Material and Methods

We performed a systematic search of the literature indexed on PubMed (up to 31 January 2023), using a combination of keywords and text words represented by “superinfected endometriosis” and “abscess endometriosis”. Two reviewers (M.F. and M.B.) independently screened titles and abstracts of the records that were retrieved through database searches. No language filters were applied. We also performed a manual search to include additional relevant articles, using the reference lists of key articles. Full texts of records recommended by at least one reviewer were screened independently by the same two reviewers and assessed for inclusion in the systematic review. Disagreements between the reviewers were solved by consensus. Data selection and extraction were conducted following PICOS (Population, Intervention, Comparison, Outcome, Study type) using a piloted form specifically designed for capturing information on the study and population characteristics. Data were extracted independently by two authors to ensure accuracy and consistency. The authors are experienced in systematic reviews [25,26,27,28,29,30]. Written informed consent was obtained from the patient to publish any accompanying images.

## 3. Results

### 3.1. Case Report

**Figure 1:**
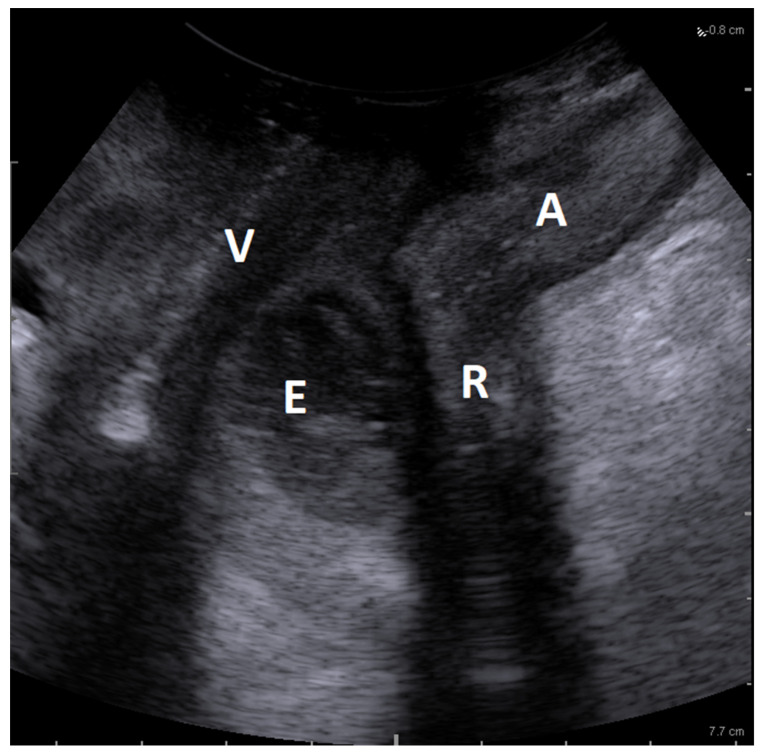
Translabial midsagittal sonographic view of the infected rectovaginal endometriosis, using a standard 2–5 MHz convex probe. Ultrasound evaluation showed an inhomogeneous iso-hypoechoic 3–4 cm lesion involving the rectovaginal septum, strictly connected to the rectal wall. V = vagina; A = anum; R = rectum; E = superinfected endometriosis.

**Figure 2:**
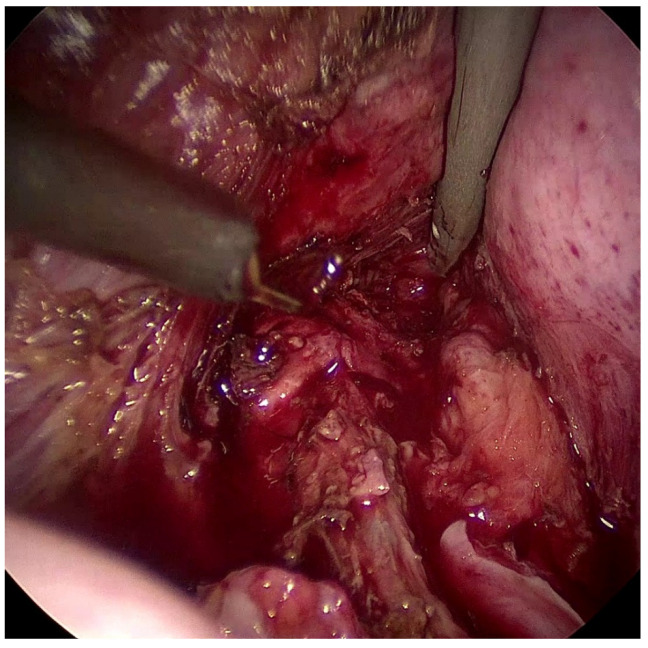
Laparoscopic view of the rectovaginal endometriosis pouch after drainage.

A 24-year-old woman was referred to our Pelvic Floor Unit for rectal–perineal pain, dyspareunia, and recurrent episodes of dense purulent vaginal discharge for one year. She was affected by diabetes mellitus type 2 in treatment with acarbose. She previously underwent multiple gynecological visits for recurrent pain and fever, describing a recurrent abscess of rectovaginal space with purulent material spilling during manual squeezing. The culture of the purulent material revealed Streptococcus Agalactiae colonization. Magnetic resonance reported the presence of an abscess with a suspected fistulization with the rectum. Our evaluation confirmed a tender tumefaction involving the posterior vaginal wall, extended for 4 cm, but no material spill from the vaginal wall was observed. Pelvic floor ultrasound evaluation was performed with BK Medical Flex Focus 400 equipped with a standard 2–5 MHz convex probe and an endovaginal biplanar probe (BK Medical probe 8848, BK Ultrasound, Burlington, MA, USA). As previously described, with the probe in a neutral position, the linear transducer was used to obtain a 65 mm longitudinal sagittal view. Both transperineal (Figure 1) and transvaginal (Figure 2) approaches revealed an inhomogeneous iso-hypoechoic 3–4 cm lesion involving the rectovaginal septum with hyperechoic debris and peripheral positive color/power Doppler intensities. Moreover, sonographic assessment demonstrated the preservation of the integrity of the rectal wall without rectal fistulization.

After proper counseling, she was admitted to laparoscopic surgery to drain the abscess. Multiple retractions were found at the level of the peritoneum of the left uterosacral ligament. The rectovaginal pouch was thickened, involved in adhesions, and tenaciously adherent due to endometriosis. The rectum and sigmoid colon were stretched at the level of the left uterosacral and retrocervical region. Multiple small brownish peritoneal lesions referred to endometriosis were widespread in the pelvic peritoneum. The peritoneum of the left broad ligament was opened and the Kobayashi space was developed to visualize and lateralize the ureteral course. The medial pararectal spaces were developed bilaterally to isolate the rectum. The rectovaginal reflection was then incised, and the rectovaginal space was dissected until the well-known collection of the rectus–vaginal septum was opened, and the infected rectovaginal endometriosis was drained (Figure 2). The removal of the remaining significant endometriosis lesions was performed. Rectal integrity was checked through a pneumatic test. Abundant washing and drainage placement in the rectovaginal pouch completed the procedure. The surgical procedure was completed in 120 min, and blood loss was less than 100 mL. No surgical complications were observed.

The histological analysis confirmed the diagnosis of endometriosis associated with evidence of chronic inflammation, and typical epithelial and stromal components were found. The patient was successfully discharged home on postoperative day 1. After discharge, the patient started a progestogen-only pill. After one year, the patient is still asymptomatic, and there are no clinical or instrumental signs of recurrence.

### 3.2. Literature Review

The electronic database search provided a total of 286 results. After the title screening, 36 papers remained. Of them, 26 were irrelevant to the review based on title and abstract screening. Ten studies were considered for full-text assessment, met the inclusion criteria, and were incorporated into the systematic review [31,32,33,34,35,36,37,38,39,40]. No paper was added through reference list searching. All the included studies were case reports and small retrospective case series published from January 1988 to January 2023, describing 10 patients.

The main characteristics of these studies are listed in Table 1. In most cases, the patients were of childbearing age, with the median age at presentation of 40 years old (range 28–56), while one patient was in perimenopause [32], and another one was in postmenopause [35]. Two patients were 24 weeks and 17 weeks pregnant [34,38]. In eight cases, extra-ovarian endometriosis abscesses were found [31,33,34,36,37,38,39,40], while in two cases, the abscesses derived from adenomyosis [32,35]. The extra-ovarian endometriosis was, respectively, in the kidney [33], in the appendix [34,38], within the pelvirectal/supralevator space [31], in the vescico-uterine pouch [36], in the cervix [37], in the liver [39], and in the psoas muscle [40]. In particular, both pregnant patients developed acute appendicitis with appendiceal endometriosis, one associated with a peritoneo-vaginal fistula [34,38]. The leading symptom was abdominal or pelvic pain (for eight patients) [31,32,33,34,36,38,39,40]; two patients also had fever [33,34], and one had night sweats/hot flashes [35]. One patient had no symptoms and was investigated for a large cervical mass, suspected of malignancy [37]. Ultrasound resulted in being the imaging tool of choice in most cases, followed by computerized tomography and magnetic resonance. Detected microorganism species resulted in a very heterogeneous population, including bacteria and amoeba. In two cases, the first-line approach was conservative with antibiotics [36,38]. However, it did not succeed in both cases, and the patients underwent surgery. In most cases, the main treatment option was the surgical one, with the resection of the endometriosis lesions [33,34,39,40] with eventual appendicectomy [34,38] or hysterectomy (with or without bilateral salpingo-oophorectomy) [31,32,35,37]. In one case, the drainage of the lesion was performed [36].

## 4. Discussion

Although not all women with endometriosis are symptomatic, endometriosis-associated pain represents the main clinical hallmarks of this disease. Pain has been shown to negatively affect the quality of life in a wide range of activities and life domains such as physical functioning; everyday activities and social life; education and work; sex, intimacy, and intimate partnerships; and mental health and emotional well-being [41]. Pain relief represents one of the main goals in treating endometriosis, and different options are available, including analgesics, combined hormonal contraceptives, progestogens, GnRH agonists and antagonists, aromatase inhibitors, and surgery. However, pain can originate from a series of different endometriosis-related conditions, and this can affect the effectiveness of the treatment. Specifically, the superinfection of endometriosis represents a rare event, and there are no specific guidelines for the treatment of this complication.

To the best of our knowledge, this is the first review focusing on the superinfection of extra-ovarian endometriosis. However, certain limitations should be stated. Since this is a rare condition, the population of interest is, as a matter of course, small. Moreover, this literature review mainly relies on isolated case reports, thus being subjected to publication bias. However, we could describe the epidemiology, presentation, and management of the superinfection of extra-ovarian endometriosis. Moreover, we also presented a case report of a 24-year-old woman in which a recurrent abscess of a rectovaginal septum led to the diagnosis of endometriosis. Pelvic floor ultrasound was able to characterize this lesion, which was managed through laparoscopic drainage.

### 4.1. Presentation

The presentation of this condition is variable, so the diagnosis can be difficult and delayed. In our review of the literature, the most reported symptom was abdominal or pelvic pain, while only three patients had a fever or night sweats/hot flashes as evidence of the inflammatory/infectious disease. The inconstant presence of classic signs of infection can make the recognition of the endometriosis superinfection more complicated and delayed. In addition, one patient was asymptomatic and investigated for a large cervical mass, with suspicion of malignancy.

### 4.2. Etiology

The mechanism through which endometriosis lesions get superinfected is not well defined. Potential pathogenetic mechanisms involve direct inoculation, ascension via the lower genital tract, translocation from the adjacent organs (such as the bowel and the vagina), and hematogenous/lymphatic spread [24]. In our case, Streptococcus Agalactiae was identified, suggesting translocation from the vagina. In the literature review, the detected microorganism species resulted in a heterogeneous population, including bacteria—mostly anaerobic—and amoeba. However, in half of the cases, a specific pathogen was not identified. This makes it more difficult to hypothesize a reproducible etiopathogenetic pattern for the development of abscesses in the context of endometriosis lesions. The propensity of endometriomas to become infected has been related to locally reduced resistance to infection as well as to endometriotic blood debris acting as an effective culture medium [22]. Of note, according to our review, superinfection occurred in 20% of cases in pregnant patients. Probably, hormonal and immune system modulation during pregnancy contributes to predisposing endometriosis lesions to superinfection. In these cases, acute appendicitis with appendiceal endometriosis was found. This represents a very rare condition during pregnancy, which ranges between 3 and 8 deliveries per 10,000 [42]. The etiopathogenetic mechanisms may involve the progestin-induced decidualization of the endometrial stromal cells which can also affect ectopic stromal endometrial cells. Deciduosis of the appendix is histologically identified at the serosa of the appendix due to the physiological reaction of the pluripotential submesothelial stromal cells to the hormonal influences of pregnancy [43] or, as reported by Dogan et al., with evident endometrial glands demonstrated by cytokeratin 7 immunohistochemistry detection [34]. Moreover, during pregnancy, the immune system protects itself from rejecting foreign paternal antigens by altering the Th1/Th2 cytokine level to Th2 cytokine dominance [44], which covers the fetus from the Th1-mediated immune system at the fetal–maternal interface. Nevertheless, this also makes pregnant women susceptible to infections with a Th1-dependent immune response [44].

### 4.3. Imaging

Different diagnostic tools have been used in the considered cases of superinfected endometriosis, including traditional X-ray radiology, CT scan, magnetic resonance, and ultrasound. However, the latter emerged as the preferred instrumental tool for assessing superinfected endometriosis.

Ultrasound is a well-established and effective instrumental tool to detect and characterize endometriosis lesions. However, variances in the appearance of endometriosis lesions should be accounted for, depending on the anatomic site and the presence of fibrosis [45]. Lesions usually appear as nodular or flat hypoechoic lesions without color Doppler spots. The sonographic identification of the superinfection of endometriosis may not be easy, and diagnosis should be made while also considering clinical and biochemical data. Typical aspects involve a spherical or oblong structure largely anechoic or hypoechoic with hyperechoic debris. Moreover, positive color/power Doppler intensities suggest abscess transformation [46].

**Figure 3:**
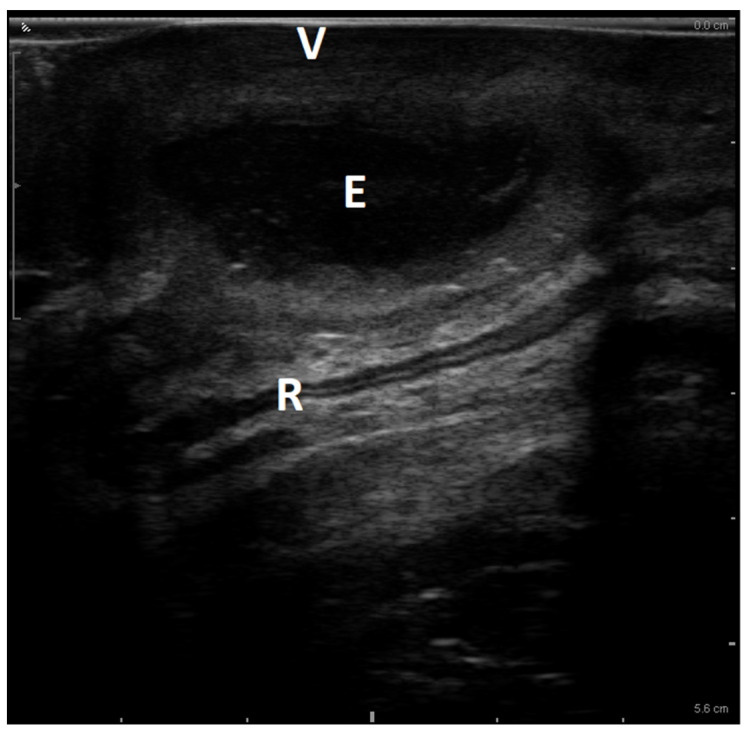
Transvaginal midsagittal sonographic view of the infected rectovaginal endometriosis, using an endocavitary linear probe. Ultrasound evaluation showed an inhomogeneous iso-hypoechoic 3–4 cm lesion involving the rectovaginal septum, but the integrity of the rectal wall is demonstrated and no fistula tract is observed. V = Vagina; R = Rectum; E = superinfected endometriosis.

In our case report, pelvic floor ultrasound was the diagnostic of choice due to the potential to investigate the rectovaginal space [47] optimally. Pelvic floor ultrasonography has recently emerged as a new diagnostic tool and has demonstrated many advantages compared to traditional imaging, including cost-effectiveness, relative ease of use, and wide availability in clinical settings [48]. In particular, pelvic floor ultrasonography has been successfully applied to the study of connective structures in different conditions, such as stress urinary incontinence and pelvic organ prolapse [49,50]. Rectovaginal space can be evaluated either with translabial (Figure 1) or transvaginal (Figure 3) approaches. Transvaginal linear probes have some potential advantages, such as a lack of geometrical distortion and a more excellent resolution of the layers of the rectovaginal space [48].

## 5. Management

In none of the considered cases did conservative management alone successfully treat endometriosis superinfection. Percutaneous drainage with interventional radiology represented a feasible mini-invasive option. However, the surgical method of choice in most cases was traditional surgery, primarily by the open abdominal route. Conventional surgery may offer some potential advantages compared to percutaneous drainage, including the possibility of evaluating the whole pelvis and abdominal cavity; to perform more efficient drainage and washing of all the purulent content; and to treat any associated endometriosis lesions. However—when feasible—the laparoscopic approach should be preferred, due to lower pain and a faster return to activities. In our case, laparoscopy allowed us to successfully drain the abscess, perform adhesiolysis, and remove all the significant endometriosis lesions in the pelvis. Moreover, no complications occurred, and early discharge home was performed.

## 6. Conclusions

In conclusion, in the case of pelvic abscess, the superinfection of endometriosis lesions should be suspected, and this can represent the onset symptom of endometriosis. Ultrasonography may show nodular or flat hypoechoic lesions with hyperechoic debris and peripheral positive color/power Doppler intensities. The goal of management is to drain the abscess, either percutaneously or via traditional surgery, followed by proper hormonal therapy to reduce recurrence.

## Figures and Tables

**Table 1:** Main characteristics of the studies included in the systematic review.

First Author and Ref.	Year and Country	Age and Previous Clinical History	Symptoms and Physical Examination	Instrumental Diagnostic	Diagnosis and Bacterium Detection	Management and Follow Up
Anderson [31]	1988, USA	32 yy, Several incisions and drainages of recurrent supraelevator abscesses	Lower quadrant abdominal pain. Tenderness of the abdomen in the tender lower quadrants. Hard, nontender, indurated mass at 8 cm on the posterior fourchette	X-ray and CT: distended bowel loops numerous large multicystic masses occupying the entire true pelvis	Pelvic endometriosis abscess. Detected Klebsiella species	LPT: Supracervical hysterectomy, bilateral salpingo-oophorectomy, sigmoid loop colostomy, and appendicectomy performed. The colostomy was closed two months after surgery. No recurrence (two years follow-up)
Bulut [32]	2013, Turkey	56 yy	Menorrhagia after three months of amenorrhea, pelvic and lumbar pain. Lobulated hard masses filling the pelvis up to the umbilicus	US: well-defined nodular masses around the uterus MRI: giant hypervascular lobulated mass of 8 × 12 × 13 cm, with central necrosis	Abscessed uterine mass and extrauterine adenomyomas with uterus-like features. Negative colture	LPT: total hysterectomy and bilateral salpingo-oophorectomy with excision of intraligamentary bilateral leiomyoma-like masses and intraperitoneal adhesions. The postoperative period was uneventful
Cheng [33]	2015, Taiwan	53 yy, Acute pyelonephritis in the past	Intermittent recurrent right flank pain, mild fever for several years. Bilateral costovertebral angle tenderness	X-ray: bilateral renal stones. US: contracted right kidney. CT: kidney abscess with the invasion of psoas muscle.	Endometriosis in a kidney with xanthogranulomatous pyelonephritis and a perinephric abscess. Detected Citrobacter koseri	CT: percutaneous drainage followed by right nephrectomy 3 days later. Asymptomatic at one month follow-up visit
Dogan [34]	2012, Turkey	30 yy	Fever, nausea, right lower quadrant pain at 24 weeks of pregnancy; left lower quadrant pain, high fever and vomiting at 28 weeks. Tenderness on abdominal examination and rebound tenderness at the right side of the uterus	At 24 weeks US: pericecal fluid At 28 weeks US: a 5 cm complicated left ovarian cystic mass compatible with tubo-ovarian abscess	At 24 weeks: endometriosis and deciduosis of appendix and acute appendicitis; at 28 weeks: infected endometrioma with deciduosis. Colture n/A.	At 24 weeks LPT: appendicectomy At 28 weeks LPT abscess was drained and left salpingectomy was performed. On postoperative day 5, the patient gave birth to a 1400 gr healthy male baby with spontaneous vaginal delivery.
Erguvan [35]	2003, Turkey	54 yy	Inguinal pain, night sweats, hot flashes. Irregularly enlarged uterus	Transvaginal US and MRI: 95 × 84 mm leiomyoma-like lesion. MRI: intramural uterine leiomyoma with cystic and necrotic degenerations	Histopathological diagnosis: abscess formation arising in a focus of adenomyosis. Colture n/A	Endometrial biopsy: inadequate. LPT to exclude malignancy: total abdominal hysterectomy and bilateral salpingo-oophorectomy. The postoperative period was uneventful
Matsuda [36]	2021, Japan	31 yy, Dysmenorrhea when she was 16 years old. Diagnosis of cystic lesion in the vescico-uterine pouch	Severe abdominal pain after oocyte retrieval. Before the oocyte retrieval: a goose-egg-sized anteverted and anteflexed uterus. Uterine mobility was low, and pain due to pressure on the uterus	Transvaginal US and MRI 4 cm cystic lesion low-absorbing similar to an abscess in the vescico-uterine pouch	Abscessed cystic endometriosis lesion in the vescico-uterine pouch. Detected Prevotella bivia.	Failure of conservative therapy, followed by LPS surgery: drainage of the abscess and ablation of the cystic lesion. She was discharged home on the same day. Embryo transfer was performed 3 months after surgery, resulting in pregnancy
Oda [37]	2014, Japan	41 yy, previous multiple myomectomy with opening of the anterior endocervical canal	Asymptomatic (she had undergone myomectomy of multiple uterine fibroids 13 years previously). Large cervical mass	Tranvaginal US: multilocular, hypoechoic lesions, with heterogeneous internal echogenicity. MRI: irregularly shaped cystic mass (overall diameter, >15 cm) in the upper anterior cervix	Histopathological diagnosis: Pyogenic cervical cyst exhibiting signs of endometriosis. Detected Escherichia Coli	LPT: total hysterectomy
Saleh [38]	2020, USA	36 yy	Lower abdominal pain, loss of appetite at 17 weeks of pregnancy. Tachycardia, spontaneous rupture of yellow purulent fluid from the posterior vaginal fornix, sepsis 24 h later	Transvaginal US: 5 × 2 cm multiloculated abscess in the posterior cul-de-sac MRI: right ovarian torsion versus abscess formation due to appendicitis	Histopathological diagnosis: abscess of endometriosis of the appendix with peritoneovaginal fistula. Colture n/A.	Failure of conservative therapy followed by LPS surgery (appendicectomy and toilette). Uncomplicated normal spontaneous vaginal delivery at 40 weeks
Sopha [39]	2015, USA	47 yy, Long history of diarrhea	Diarrhea, acute right upper quadrant and back pain with nausea and vomiting	CT: liver abscess	Histopathological diagnosis: Abscess of hepatic uterus-like mass. Detected Aeromonas sp, Entamoeba histolytica	Antibiotic therapy for two weeks followed by a CT-guided fine-needle aspiration and LPS surgery: segment VII excisional biopsy. Asymptomatic on follow-up evaluation
Zhao [40]	2018, China	28 yy	Eight-month history of lower abdominal and back pain and swelling	US: a cystic mass of 12 cm rising from the iliopsoas or left ureter. CT: suspect psoas abscess with left hydronephrosis MRI: A 10 cm mass near the left iliac vessel constraining the left ureter, left retroperitoneal endometriosis, adenomyosis	Histopathological diagnosis: psoas muscle endometriosis abscess. Colture n/A.	Left ureteral stenting and percutaneous drainage under local anesthesia, antibiotic administration. GnRH treatment for three months followed by LPS surgery: resection of the psoas muscle endometriosis. Asymptomatic at three-month follow-up.

Abbreviations: US, ultrasonography; MRI, magnetic resonance imaging; CT, computerized tomography; LPT laparotomy, LPS laparoscopy, GFR: glomerular filtration rate; yy years.

## Data Availability

Data sharing not applicable.
